# Associations between digital media use behaviours, screen time and positive mental health in youth: results from the 2019 Canadian Health Survey on Children and Youth

**DOI:** 10.1186/s12889-025-22874-2

**Published:** 2025-07-03

**Authors:** Zahra M. Clayborne, Colin A. Capaldi, Vrati M. Mehra

**Affiliations:** 1 Centre for Surveillance and Applied Research, Public Health Agency of Canada, 785 Carling Ave, Ottawa, ON K1A 0K9 Canada; 2 Department of Pediatrics, Cumming School of Medicine, University of Calgary, Calgary, AB Canada; 3 Temerty Faculty of Medicine, University of Toronto, Toronto, ON Canada

**Keywords:** Positive mental health, Digital media use, Social media, Video games, Screen time, Sedentary behaviour, Population health, Adolescence

## Abstract

**Background:**

Limited research has examined associations between a range of digital media use (DMU) behaviours and screen time measures with positive mental health (PMH) outcomes among Canadian adolescents. This study examined these associations among a large sample of Canadian youth.

**Methods:**

We used self-reported data from youth aged 12–17 years in the 2019 Canadian Health Survey on Children and Youth (*N* = 10,695). DMU behaviours included frequency of using social media, video/instant messaging and online gaming. Screen time included estimated hours spent watching content, playing video games and overall sedentary electronic device usage in the past week. PMH outcomes included self-rated mental health (SRMH), life satisfaction, happiness, autonomy, competence and relatedness. We conducted gender-stratified adjusted logistic regression analyses.

**Results:**

Girls reporting using social media constantly (vs. never or less than weekly) were less likely to report high SRMH, life satisfaction, happiness, autonomy and competence, while their video/instant messaging frequency was unrelated to PMH outcomes. Social media use and video/instant messaging frequency tended to be unrelated to PMH outcomes among boys (or positively associated at moderate levels in a few exceptions). Boys and girls reporting online gaming constantly (vs. never or less than weekly) were less likely to report high happiness, autonomy, competence and relatedness. Boys watching content for 14+ (vs. < 3) hours in the past week had lower odds of high SRMH, life satisfaction and happiness, while girls watching 7+ (vs. < 3) hours had lower odds of all PMH outcomes. Boys and girls reporting 21+ (vs. < 3) hours of overall sedentary electronic device use in the past week had lower odds of all PMH outcomes.

**Conclusions:**

This study provides support for a link between some DMU behaviours and screen time measures with lower PMH among Canadian youth. Findings can assist in the promotion of public health strategies targeted towards promoting reduced and/or non-problematic DMU and screen time, and improved well-being, as well as the continued surveillance of DMU, screen time and PMH outcomes among Canadian youth.

**Supplementary Information:**

The online version contains supplementary material available at 10.1186/s12889-025-22874-2.

## Background

The use of electronic devices (e.g. smartphones, tablets) and associated digital media use (DMU) behaviours (e.g. online gaming, social media use [SMU], video/instant messaging) have become commonplace in the lives of young people. In a MediaSmarts survey from 2021, most youth (12–17 year-old) respondents in Canada reported having their own smartphone [1]. Furthermore, in 2019, the majority of youth in Canada reported using social media and video/instant messaging, and around a fifth reported playing online video games, at least several times per day [2].

Given this context, it is perhaps unsurprising that many young people are spending a lot of time in front of screens. While DMU specifically involves “content transmitted over the Internet or computer networks” [3], p.402, screen time (ST) is defined as “time spent with any screen, including smart phones, tablets, television, video games, computers, or wearable technology” [3], p.402. According to the Public Health Agency of Canada’s Physical Activity, Sedentary Behaviour and Sleep Indicators, approximately half of individuals aged 5–17 years in 2018–2019 did not meet the guideline of no more than 2 hours of recreational ST per day [4, 5]. Moreover, recreational ST appears to have been increasing over time among youth in Canada before the COVID-19 pandemic [6], with further increases observed during the pandemic [7]. 

ST and DMU have been identified by some as playing a potential role in worsening youth mental health [8, 9, 10]. Recent meta-analytic research tends to find support for an association between SMU and ill-being among youth, although there is considerable variability across studies and associations can be very small [11]. Longitudinal studies provide some evidence for total ST predicting subsequent depressive symptoms; nevertheless, associations are also small in magnitude, with limited evidence for some types of ST (i.e. television/video use and video games) and other internalizing symptoms (i.e. anxiety) [12].

Importantly, mental health does not only comprise the absence of distress or a mental disorder; the presence of well-being or positive mental health (PMH) is essential for complete mental health [13, 14]. While meta-analyses have reported null associations between SMU and PMH for youth, [15, 16] some studies suggest that the relationship in this age group may depend on various factors, such as gender, the wider societal prevalence of intense SMU, whether the use is problematic (i.e. addiction-like), whether it is being used actively or passively, and individual differences in susceptibility [17, 18, 19, 20]. The associations of PMH with other types of DMU and ST also appear to be nuanced [3]. For instance, some research finds support for nonlinear associations between PMH and free time spent playing video games, watching content, and using computers or smartphones, with ST tending to only be associated with lower PMH above certain thresholds [21, 22].

Findings among youth in Canada have been somewhat mixed. Fewer after-school hours of ST was associated with higher life satisfaction among grade 7 students in British Columbia, [23] but meeting ST recommendations was not significantly associated with high self-rated mental health (SRMH) in Canadian youth [24]. A study using data from the 2019 Canadian Health Survey on Children and Youth (CHSCY) found that girls who engaged in less frequent SMU and video/instant messaging, and did not play online video games tended to be more likely to report higher SRMH, while the frequency of these DMU behaviours was largely unrelated to SRMH for boys [2]. This study also investigated associations with negative psychological outcomes (e.g. suicidal ideation), [2] but did not examine the numerous additional PMH outcomes or measures of ST that were also captured in the 2019 CHSCY. To fill this gap, we examined gender-stratified associations between frequency of DMU behaviours (i.e. SMU, video and instant messaging, online gaming) and ST duration (i.e. time spent watching content, gaming or using electronic/media devices) with six indicators of PMH in a sample of youth aged 12–17 years from the 2019 CHSCY. We hypothesized that youth who engaged in more frequent DMU behaviours and longer ST would report lower PMH.

## Methods

### Data and participants

We analyzed cross-sectional data from the 2019 CHSCY, which were collected between 11 February–2 August 2019 from individuals aged 1–17 years living in Canada’s 10 provinces and 3 territories [25]. The 2019 CHSCY used the Canadian Child Benefit as its sampling frame. Survey coverage represented approximately 98% and 96% of children and youth aged 1–17 years in the provinces and territories, respectively. Those who were institutionalized, living in foster homes, or living on First Nations reserves/settlements were excluded from the survey. The 2019 CHSCY was voluntary and could be completed online or by telephone. Analyses were restricted to youth (aged 12–17 years; response rate = 41.3%) to align with the youth Positive Mental Health Surveillance Indicator Framework [26]. As this dataset was available through a data sharing agreement between the Public Health Agency of Canada and Statistics Canada, research ethics board approval for this study was not required. We had access to data from 11,077 out of the 13,602 (81.4%) youth respondents who agreed to share their data. Informed assent and consent were obtained from all participants, and consent from parents/guardians was also obtained for respondents aged 12–14 years.

### Measures

#### Digital media use

Youth were asked “How often do you go online for the following activities?”, with social networking (e.g. Facebook, Instagram or Twitter; labelled “social media” hereafter), video or instant messaging (e.g. WhatsApp, Snapchat or FaceTime) and online gaming (e.g. League of Legends, Minecraft or World of Warcraft) listed as activities. Youth rated their frequency of engaging in each from “Never” to “Constantly”. While a previous study recoded the response options into three categories, [2] we only combined the two lowest response options (i.e., “Never” and “Less than weekly”) as we were interested in determining how frequent each DMU had to be before it was linked to lower (or before it was not linked to higher) PMH.

#### Screen time

Youth were asked “In the past 7 days, how much time did you spend doing the following?”, with watching content (movies, videos, Youtube, Netflix, or television programs), playing video games (using any console or electronic device) and using any electronic or media device (mobile device, computer, tablet, video game console, or television while sitting or lying down; excluding time spent in class, doing homework or reading for enjoyment) listed as activities. Youth rated the amount of time they engaged in each using response options that ranged from “No time” to “21 hours or more”. Due to small cell sizes, the “No time” category was collapsed with the “Less than 3 hours” category for analyses. Similar to the DMU variables, the remaining response categories of the ST variables were not collapsed so that we could investigate detailed thresholds of exposure that were (or were not) linked to PMH.

#### Positive mental health

SRMH was assessed by asking “In general how is your mental health?”. Five response options ranged from “Excellent” to “Poor”. While a previous study classified those who responded “Good”, “Very good” or “Excellent” as having higher SRMH, [2] we categorized “Excellent” or “Very good” as high SRMH to be consistent with how this measure is typically coded in PMH surveillance [26].

Life satisfaction was assessed by asking “Using a scale of 0 to 10, where 0 means ‘very dissatisfied’ and 10 means ‘very satisfied’, how do you feel about your life as a whole right now?”. Responses of 9 or 10 were categorized as high life satisfaction [26].

Happiness was assessed by asking “How would you usually describe yourself?”. Five response options ranged from “Happy and interested in life” to “So unhappy that life is not worthwhile”. Responding “Happy and interested in life” was categorized as high happiness [26].

Aspects of psychological and social well-being were assessed using the Children’s Intrinsic Needs Satisfaction Scale (CINSS) [27]. Six items assessed autonomy (e.g. “I feel free to express myself with my friends”), six assessed competence (e.g. “I feel I do things well at home”) and six assessed relatedness (e.g. “I like to be with my teachers”) with peers, at home and at school. Response options ranged from “1: Really false for me” to “4: Really true for me”. A mean score of 3 + on each dimension was coded as high autonomy, competence or relatedness [26]. Research has validated the CINSS in the 2019 CHSCY [28].

#### Sociodemographic characteristics

Several sociodemographic variables were included as covariates in adjusted logistic regression analyses, including age, population group, household income and place of residence. For population group, we coded youth as racialized if they were classified by Statistics Canada as a visible minority; [29] we coded youth as Indigenous if they identified as First Nations, Métis or Inuk/Inuit; [30] and we coded the remaining youth as non-racialized and non-Indigenous. Household income was either reported by the person-most-knowledgeable (a parent usually) or imputed by Statistics Canada; we controlled for household income quintile in particular in the adjusted analyses. Place of residence (population centre or rural area) was determined by Statistics Canada based on postal code information; individuals living in continuously built-up areas with population concentrations of 1,000 + and population densities of 400 + per km^2^ were coded as living in population centres.

Analyses were stratified by gender, which was measured by asking “What is your gender?”. Response options included “Male”, “Female”, and “Or please specify”. We refer to those who answered “male” as “boys” and “female” as “girls”. Results for non-binary youth are not reported as their sample size was small in the 2019 CHSCY [31].

### Analyses

Statistical analyses were conducted using SAS Enterprise Guide version 7.1 (SAS Institute, Cary, NC, USA). To account for the complex survey design, non-response and non-sharing, all estimates were weighted using sampling weights provided by Statistics Canada and variances were estimated using bootstrap weights. Descriptive statistics were reported using weighted percentages with 95% confidence intervals (CIs).

Adjusted logistic regression analyses were conducted to examine the associations between the DMU and ST variables with each PMH outcome. The first set of adjusted regression analyses included self-reported frequency of SMU, video/instant messaging and online gaming as explanatory variables. The second set included self-reported hours spent watching content and playing video games in the past seven days. The third included self-reported hours of sedentary electronic device use in the past seven days as the explanatory variable. A separate regression analysis for overall electronic device usage was conducted as it captures sedentary ST regardless of the specific activity being engaged in on the device, but could include watching content and playing video games. Sociodemographic characteristics were included as covariates in adjusted analyses. Unadjusted logistic regression analyses were also conducted and are reported in the Supplementary File. Results were reported using odds ratios with 95% CIs. Statistical significance was ascertained by 95% CIs that did not include 1.00. Descriptive statistics and logistic regression analyses were conducted separately for boys and girls. The logistic regression analyses only included youth with data on all variables of interest, which represented 97.0% of boy and girl respondents.

## Results

Gender-stratified distributions of the DMU, ST, PMH and sociodemographic variables are reported in Table 1. There were gender differences in many of the main variables. For instance, boys tended to report engaging in online gaming more frequently, while girls tended to report engaging in video/instant messaging and social media more frequently. Moreover, high SRMH, life satisfaction and happiness were more commonly reported by boys than girls.

**Table 1:** Gender-stratified descriptive statistics for digital media use, screen time, positive mental health and sociodemographic variables

Variables	Boys (*N* = 5,462)		Girls (*N* = 5,564)
	% (95% CI)		% (95% CI)
**Digital media use measures**			
Frequency of social media	***n*** **= 5**,**451**		***n*** **= 5**,**551**
Never or less than weekly	25.5 (24.1, 27.0)		14.3 (13.1, 15.6)
Weekly	5.2 (4.4, 6.0)		3.5 (2.8, 4.2)
Once a day	15.5 (14.2, 16.9)		11.8 (10.6, 13.0)
Several times a day	42.2 (40.6, 43.9)		50.9 (49.1, 52.7)
Constantly	11.5 (10.4, 12.6)		19.5 (18.1, 20.9)
Frequency of video/instant messaging	***n*** **= 5**,**444**		***n*** **= 5**,**547**
Never or less than weekly	33.4 (31.8, 35.1)		20.0 (18.5, 21.5)
Weekly	7.1 (6.2, 8.0)		6.5 (5.6, 7.4)
Once a day	15.5 (14.2, 16.8)		14.3 (13.0, 15.6)
Several times a day	32.6 (31.0, 34.3)		40.5 (38.8, 42.2)
Constantly	11.3 (10.3, 12.3)		18.7 (17.3, 20.1)
Frequency of online gaming	***n =*** **5**,**448**		***n*** **= 5**,**549**
Never or less than weekly	32.0 (30.3, 33.8)		80.9 (79.4, 82.3)
Weekly	16.4 (15.0, 17.7)		6.9 (6.0, 7.9)
Once a day	21.2 (19.8, 22.6)		6.2 (5.3, 7.1)
Several times a day	20.9 (19.5, 22.3)		3.9 (3.3, 4.6)
Constantly	9.5 (8.5, 10.5)		2.1 (1.6, 2.5)
**Screen time measures**			
Hours watching video content in past week	***n =*** **5**,**446**		***n*** **= 5**,**548**
< 3 h	15.0 (13.7, 16.3)		16.1 (14.8, 17.5)
3 to < 7 h	30.6 (29.0, 32.2)		32.3 (30.6, 34.0)
7 to < 14 h	25.3 (23.8, 26.8)		25.7 (24.1, 27.3)
14 to < 21 h	16.7 (15.3, 18.0)		14.8 (13.5, 16.0)
21 + hours	12.4 (11.4, 13.5)		11.2 (10.0, 12.3)
Hours playing video games in past week	***n =*** **5**,**436**		***n*** **= 5**,**525**
< 3 h	31.5 (29.8, 33.2)		71.3 (69.5, 73.0)
3 to < 7 h	24.1 (22.6, 25.6)		14.5 (13.1, 15.9)
7 to < 14 h	21.9 (20.5, 23.4)		7.7 (6.7, 8.8)
14 to < 21 h	11.6 (10.5, 12.7)		3.6 (2.9, 4.3)
21 + hours	10.9 (9.8, 12.0)		2.9 (2.3, 3.5)
Hours of sedentary electronic device use in past week	***n =*** **5**,**451**		***n*** **= 5**,**548**
< 3 h	10.0 (8.9, 11.0)		10.7 (9.6, 11.8)
3 to < 7 h	21.4 (20.0, 22.9)		24.1 (22.5, 25.6)
7 to < 14 h	22.0 (20.5, 23.4)		26.0 (24.4, 27.6)
14 to < 21 h	21.9 (20.4, 23.4)		19.7 (18.2, 21.2)
21 + hours	24.8 (23.3, 26.3)		19.6 (18.1, 21.1)
**Positive mental health measures**			
High self-rated mental health	***n =*** **5**,**441**		***n*** **= 5**,**547**
	73.7 (72.2, 75.2)		58.7 (57.0, 60.4)
High life satisfaction	***n =*** **5**,**455**		***n*** **= 5**,**554**
	49.0 (47.2, 50.8)		41.1 (39.3, 43.0)
High happiness	***n =*** **5**,**450**		***n*** **= 5**,**555**
	68.1 (66.5, 69.8)		60.6 (58.8, 62.3)
High autonomy	***n =*** **5**,**440**		***n*** **= 5**,**549**
	81.5 (80.2, 82.9)		80.3 (78.9, 81.7)
High competence	***n =*** **5**,**437**		***n*** **= 5**,**550**
	85.3 (84.0, 86.7)		83.5 (82.1, 84.9)
High relatedness	***n =*** **5**,**436**		***n*** **= 5**,**551**
	89.2 (88.1, 90.3)		89.7 (88.6, 90.9)
**Sociodemographic characteristics**			
Age in years	***n*** **= 5**,**462**		***n*** **= 5**,**564**
12	18.4 (17.1, 19.7)		18.0 (16.6, 19.5)
13	16.6 (15.3, 18.0)		16.6 (15.3, 18.0)
14	16.5 (15.2, 17.9)		16.0 (14.7, 17.4)
15	18.0 (16.6, 19.4)		16.5 (15.2, 17.9)
16	15.7 (14.4, 17.0)		17.7 (16.2, 19.2)
17	14.8 (13.4, 16.1)		15.1 (13.8, 16.3)
Population group	***n*** **= 5**,**449**		***n*** **= 5**,**537**
Non-racialized and non-Indigenous	66.7 (65.1, 68.3)		65.3 (63.6, 66.9)
Racialized	28.6 (27.0, 30.1)		30.1 (28.6, 31.7)
Indigenous	4.8 (4.1, 5.4)		4.6 (3.9, 5.3)
Place of residence	***n*** **= 5**,**462**		***n*** **= 5**,**564**
Population centre	81.2 (80.0, 82.5)		82.3 (81.1, 83.6)
Rural area	18.8 (17.5, 20.0)		17.7 (16.4, 18.9)
Household income	***n*** **= 5**,**462**		***n*** **= 5**,**564**
Median household income (CAD)	95,896 (92,346.8, 99,445.3)		92,954 (90,219.0, 95,688.8)

**Source**: 2019 Canadian Health Survey on Children and Youth

**Abbreviations**: CAD, Canadian dollars; CI, confidence interval

**Notes**: Samples sizes are unweighted; all other values are weighted

### Digital media use and positive mental health

Adjusted associations between DMU behaviours and PMH outcomes are reported in Table 2 for boys and Table 3 for girls; unadjusted associations are reported in Table S1 for boys and Table S2 for girls in the Supplementary File. We focus our reporting on associations that were significant in the adjusted analyses.

**Table 2:** Adjusted associations between digital media use behaviours and positive mental health among boys (*N* = 5,299)

	High self-rated mental health		High life satisfaction		High happiness		High autonomy		High competence		High relatedness	
	% (95% CI)	aOR (95% CI)	% (95% CI)	aOR (95% CI)	% (95% CI)	aOR (95% CI)	% (95% CI)	aOR (95% CI)	% (95% CI)	aOR (95% CI)	% (95% CI)	aOR (95% CI)
**Social media**												
Never or less than weekly	75.7 (72.7, 78.6)	Ref.	51.6 (48.1, 55.0)	Ref.	67.2 (63.9, 70.5)	Ref.	78.3 (75.6, 81.1)	Ref.	86.2 (83.9, 88.5)	Ref.	90.1 (88.1, 92.0)	Ref.
Weekly	75.5 (69.3, 81.7)	1.06 (0.71, 1.57)	52.1 (44.6, 59.6)	1.17 (0.82, 1.68)	64.2 (56.7,71.7)	0.94 (0.64, 1.37)	76.3 (69.1, 83.4)	0.80 (0.52, 1.24)	81.9 (75.4, 88.3)	0.79 (0.48, 1.31)	88.0 (82.5, 93.5)	0.85 (0.47, 1.55)
Once a day	77.5 (73.9, 81.1)	1.21 (0.92, 1.59)	49.1 (44.5, 53.8)	1.06 (0.83, 1.35)	69.0 (64.7, 73.3)	1.21 (0.93, 1.58)	84.9 (81.4, 88.3)	**1.48 ** **(1.07**,** 2.05)**	86.8 (83.2, 90.4)	1.21 (0.83, 1.78)	88.5 (85.4, 91.7)	0.90 (0.61, 1.32)
Several times a day	72.3 (69.9, 74.8)	1.07 (0.83, 1.38)	48.2 (45.5, 50.9)	1.20 (0.97, 1.49).	69.9 (67.4, 72.5)	**1.40** **(1.11**,** 1.76)**	83.4 (81.4, 85.3)	**1.37** **(1.04**,** 1.79)**	85.9 (83.9, 87.9)	1.25 (0.90, 1.74)	89.9 (88.3, 91.5)	1.16 (0.82, 1.65)
Constantly	70.3 (66.0, 74.6)	1.21 (0.85, 1.72)	44.9 (39.4, 50.5)	1.24 (0.88, 1.74)	65.1 (60.2, 69.9)	1.25 (0.88, 1.78)	80.3 (76.3, 84.3)	1.29 (0.86, 1.92)	80.9 (76.5, 85.3)	0.97 (0.61, 1.53)	85.7 (82.1, 89.4)	0.92 (0.54, 1.56)
**Video/instant messaging**												
Never or less than weekly	73.9 (71.3, 76.5)	Ref.	50.0 (46.7, 53.2)	Ref.	66.6 (63.5, 69.7)	Ref.	78.5 (76.0, 81.1)	Ref.	85.3 (83.0, 87.6)	Ref.	88.6 (86.6, 90.6)	Ref.
Weekly	81.8 (76.9, 86.7)	**1.70** **(1.18**,** 2.45)**	54.7 (47.7, 61.8)	1.31 (0.95, 1.82)	69.2 (62.3, 76.1)	1.23 (0.85, 1.78)	86.0 (81.5, 90.5)	**1.73** **(1.15**,** 2.61)**	87.5 (82.2, 92.7)	1.30 (0.76, 2.22)	89.7 (85.3, 94.2)	1.24 (0.71, 2.16)
Once a day	77.2 (73.5, 81.0)	**1.32** **(1.002**,** 1.74)**	52.2 (47.6, 56.8)	1.23 (0.96, 1.58)	71.0 (66.9, 75.1)	1.29 (0.99, 1.68)	83.9 (80.7, 87.2)	**1.35** **(1.0002**,** 1.81)**	85.8 (82.4, 89.3)	1.14 (0.79, 1.65)	91.9 (89.5, 94.3)	**1.70** **(1.14**,** 2.53)**
Several times a day	73.4 (70.6, 76.2)	1.25 (0.97, 1.60)	48.0 (44.8, 51.1)	1.12 (0.90, 1.40)	69.8 (66.9, 72.6)	**1.27** **(1.001**,** 1.60)**	83.3 (81.2, 85.5)	1.27 (0.96, 1.67)	85.9 (83.8, 88.1)	1.20 (0.86, 1.68)	89.3 (87.4, 91.2)	1.18 (0.84, 1.66)
Constantly	66.2 (61.8, 70.5)	0.92 (0.66, 1.28)	41.6 (36.6, 46.5)	0.90 (0.66, 1.24)	64.3 (59.7, 68.9)	1.11 (0.80, 1.54)	79.8 (76.0, 83.5)	1.14 (0.79, 1.65)	81.6 (77.6, 85.6)	1.12 (0.73, 1.71)	86.4 (83.2, 89.6)	1.18 (0.72, 1.95)
**Online gaming**												
Never or less than weekly	73.4 (70.7, 76.2)	Ref.	53.1 (50.0, 56.3)	Ref.	74.7 (71.9, 77.6)	Ref.	82.7 (80.4, 85.0)	Ref.	88.4 (86.4, 90.5)	Ref.	91.9 (90.4, 93.4)	Ref.
Weekly	75.1 (71.3, 79.0)	1.01 (0.78, 1.32)	47.6 (43.0, 52.2)	**0.75** **(0.60**,** 0.94)**	68.5 (64.1, 73.0)	**0.71** **(0.55**,** 0.92)**	83.2 (79.8, 86.5)	1.03 (0.77, 1.37)	85.9 (82.6, 89.3)	0.78 (0.56, 1.10)	89.0 (85.9, 92.1)	0.70 (0.48, 1.02)
Once a day	76.6 (73.4, 79.8)	1.07 (0.84, 1.35)	48.3 (44.5, 52.1)	**0.76** **(0.62**,** 0.94)**	70.2 (66.7, 73.7)	**0.75** **(0.60**,** 0.95)**	84.3 (81.5, 87.1)	1.08 (0.82, 1.42)	86.2 (83.2, 89.2)	0.76 (0.56, 1.05)	89.5 (87.1, 92.0)	**0.71** **(0.52**,** 0.98)**
Several times a day	73.4 (70.0, 76.8)	0.90 (0.72, 1.13)	47.3 (43.2, 51.3)	**0.71** **(0.57**,** 0.88)**	62.1 (58.3, 65.8)	**0.50** **(0.40**,** 0.62)**	80.6 (77.6, 83.6)	0.81 (0.63, 1.05)	82.5 (79.5, 85.5)	**0.56** **(0.41**,** 0.75)**	88.3 (85.8, 90.8)	**0.62** **(0.45**,** 0.86)**
Constantly	68.6 (63.6, 73.6)	0.76 (0.57, 1.01)	43.3 (37.3, 49.2)	**0.64** **(0.48**,** 0.85)**	55.1 (49.4, 60.8	**0.39** **(0.30**,** 0.52)**	71.5 (66.7, 76.4)	**0.52** **(0.39**,** 0.69)**	78.2 (73.6, 82.8)	**0.47** **(0.33**,** 0.67)**	81.3 (76.6, 86.0)	**0.39** **(0.26**,** 0.57)**

**Source**: 2019 Canadian Health Survey on Children and Youth

**Abbreviations**: aOR, adjusted odds ratio; CI, confidence interval; Ref., reference group

**Notes**: Sample size is unweighted; all other values are weighted. Statistically significant results are bolded. The adjusted logistic regression analyses included age, household income quintile, place of residence, population group and the other digital media use variables as covariates

**Table 3:** Adjusted associations between digital media use behaviours and positive mental health among girls (*N* = 5,396)

	High self-rated mental health		High life satisfaction		High happiness		High autonomy		High competence		High relatedness	
	% (95% CI)	aOR (95% CI)	% (95% CI)	aOR (95% CI)	% (95% CI)	aOR (95% CI)	% (95% CI)	aOR (95% CI)	% (95% CI)	aOR (95% CI)	% (95% CI)	aOR (95% CI)
**Social media**												
Never or less than weekly	73.1 (69.2, 76.9)	Ref.	61.6 (57.2, 66.0)	Ref.	74.5 (70.5, 78.5)	Ref.	83.9 (80.5, 87.3)	Ref.	88.7 (85.6, 91.9)	Ref.	93.7 (91.4, 96.0)	Ref.
Weekly	66.6 (57.2, 75.9)	0.92 (0.56, 1.51)	44.3 (34.5, 54.0)	**0.61** **(0.38**,** 0.97)**	58.1 (48.1, 68.1)	**0.53** **(0.33**,** 0.88)**	81.2 (74.2, 88.2)	0.93 (0.54, 1.61)	84.4 (77.1, 91.7)	0.79 (0.41, 1.55)	91.3 (86.3, 96.3)	0.89 (0.40, 2.00)
Once a day	62.8 (57.7, 67.9)	0.77 (0.56, 1.05)	49.3 (43.9, 54.6)	0.74 (0.54, 1.02)	65.9 (60.8, 71.0)	**0.70** **(0.50**,** 0.96)**	80.1 (75.7, 84.6)	0.79 (0.53, 1.18)	85.2 (81.2, 89.2)	0.74 (0.45, 1.21)	90.5 (87.2, 93.7)	0.73 (0.40, 1.32)
Several times a day	57.6 (55.0, 60.2)	**0.75** **(0.58**,** 0.98)**	37.1 (34.5, 39.7)	**0.53** **(0.41**,** 0.68)**	58.3 (55.7, 60.9)	**0.59** **(0.45**,** 0.78)**	80.8 (78.9, 82.8)	0.87 (0.62, 1.23)	83.1 (80.9, 85.2)	0.72 (0.47, 1.12)	89.4 (87.7, 91.1)	0.73 (0.44, 1.21)
Constantly	48.4 (44.4, 52.4)	**0.52** **(0.38**,** 0.70)**	31.4 (27.5, 35.3)	**0.41** **(0.30**,** 0.57)**	53.2 (49.1, 57.2)	**0.53** **(0.38**,** 0.74)**	75.4 (71.9, 78.8)	**0.63** **(0.43**,** 0.92)**	78.6 (75.2, 82.0)	**0.61** **(0.37**,** 0.98)**	86.4 (83.5, 89.2)	0.57 (0.32, 1.01)
**Video/instant messaging**												
Never or less than weekly	67.3 (63.6, 71.1)	Ref.	51.8 (47.6, 55.9)	Ref.	65.5 (61.6, 69.3)	Ref.	80.8 (77.8, 83.8)	Ref.	84.6 (81.6, 87.7)	Ref.	91.5 (89.3, 93.7)	Ref.
Weekly	59.5 (52.4, 66.7)	0.74 (0.51, 1.07)	44.6 (37.2, 52.1)	0.83 (0.58, 1.20)	63.9 (56.9, 71.0)	1.02 (0.72, 1.44)	81.0 (74.9, 87.2)	1.02 (0.63, 1.67)	85.1 (78.9, 91.3)	1.08 (0.59, 1.96)	90.1 (85.5, 94.7)	0.86 (0.45, 1.64)
Once a day	61.6 (56.7, 66.4)	0.92 (0.69, 1.24)	41.9 (37.1, 46.7)	0.87 (0.66, 1.16)	65.0 (60.2, 69.9)	1.25 (0.94, 1.66)	81.6 (77.8, 85.4)	1.19 (0.84, 1.68)	85.6 (82.0, 89.2)	1.31 (0.87, 1.97)	89.7 (86.5, 92.8)	1.00 (0.62, 1.59)
Several times a day	57.0 (54.2, 59.8)	0.93 (0.73, 1.18)	38.3 (35.4, 41.3)	0.98 (0.77, 1.25)	58.9 (56.1, 61.7)	1.09 (0.86, 1.38)	79.9 (77.6, 82.2)	1.11 (0.83, 1.47)	83.4 (81.1, 85.6)	1.15 (0.81, 1.64)	89.3 (87.5, 91.2)	1.03 (0.69, 1.55)
Constantly	51.8 (47.5, 56.1)	1.01 (0.75, 1.37)	34.2 (30.3, 38.2)	1.07 (0.79, 1.44)	54.0 (49.8, 58.3)	1.04 (0.77, 1.40)	78.5 (75.2, 81.7)	1.28 (0.91, 1.78)	79.3 (75.8, 82.8)	1.04 (0.68, 1.58)	87.9 (85.3, 90.6)	1.13 (0.70, 1.84)
**Online gaming**												
Never or less than weekly	58.8 (56.8, 60.8)	Ref.	41.7 (39.7, 43.8)	Ref.	61.8 (59.8, 63.7)	Ref.	81.3 (79.7, 82.9)	Ref.	84.9 (83.4, 86.4)	Ref.	90.4 (89.1, 91.6)	Ref.
Weekly	54.5 (47.4, 61.5)	0.75 (0.55, 1.02)	36.5 (29.6, 43.4)	0.73 (0.53, 1.01)	55.5 (48.4, 62.7)	0.76 (0.56, 1.03)	73.1 (66.1, 80.2)	**0.60** **(0.41**,** 0.88)**	77.2 (70.4, 84.1)	**0.58** **(0.38**,** 0.88)**	86.5 (81.3, 91.8)	0.65 (0.40, 1.06)
Once a day	66.9 (59.8, 73.9)	1.24 (0.87, 1.75)	46.2 (38.7, 53.7)	1.07 (0.76, 1.51)	59.6 (52.0, 67.2)	0.83 (0.58, 1.17)	82.3 (76.5, 88.2)	1.06 (0.70, 1.63)	81.0 (74.4, 87.5)	0.72 (0.46, 1.12)	88.0 (82.4, 93.7)	0.76 (0.43, 1.34)
Several times a day	58.8 (50.0, 67.7)	0.98 (0.66, 1.46)	36.3 (27.8, 44.8)	0.78 (0.52, 1.17)	54.2 (44.9, 63.6)	0.75 (0.49, 1.14)	72.4 (64.3, 80.5)	**0.59** **(0.38**,** 0.89)**	73.3 (65.5, 81.2)	**0.49** **(0.31**,** 0.77)**	88.1 (82.5, 93.7)	0.78 (0.43, 1.41)
Constantly	55.0 (43.5, 66.4)	0.89 (0.51, 1.55)	29.3^C^ (18.9, 39.6)	0.58 (0.33, 1.05)	42.7 (31.1, 54.3)	**0.48** **(0.28**,** 0.83)**	65.8 (55.1, 76.4)	**0.47** **(0.27**,** 0.80)**	66.9 (56.0, 77.8)	**0.40** **(0.23**,** 0.69)**	77.3 (67.8, 86.7)	**0.38** **(0.21**,** 0.72)**

**Source**: 2019 Canadian Health Survey on Children and Youth

**Abbreviations**: aOR, adjusted odds ratio; CI, confidence interval; Ref., reference group

**Notes**: Sample size is unweighted; all other values are weighted. Statistically significant results are bolded. The adjusted logistic regression analyses included age, household income quintile, place of residence, population group and the other digital media use variables as covariates

^C^ Estimate should be interpreted with caution due to high sampling variability

#### Social media

Boys who reported SMU several times a day (vs. never or less than weekly) were more likely to report high life satisfaction and autonomy. Boys who reported SMU once per day (vs. never or less than weekly) were also more likely to report high autonomy (see Table 2).

Girls who reported constant SMU (vs. never or less than weekly) were less likely to report high SRMH, life satisfaction, happiness, autonomy and competence. Girls who reported SMU several times a day (vs. never or less than weekly) were less likely to report high SRMH, life satisfaction and happiness. Girls who reported SMU once a day (vs. never or less than weekly) were less likely to report high happiness, while girls who reported weekly SMU (vs. never or less than weekly) were less likely to report high life satisfaction and happiness (see Table 3).

#### Video/instant messaging

Boys who reported weekly use of video/instant messaging (vs. never or less than weekly) were more likely to report high SRMH and autonomy. Boys who reported video/instant messaging once per day (vs. never or less than weekly) were more likely to report high SRMH, autonomy and relatedness. Finally, boys who reported video/instant messaging several times a day (vs. never or less than weekly) were more likely to report high happiness (see Table 2).

None of the adjusted associations involving video/instant messaging were significant for girls (see Table 3).

#### Online gaming

Boys who reported online gaming constantly (vs. never or less than weekly) were less likely to report high life satisfaction, happiness, autonomy, competence and relatedness. Similarly, boys who reported online gaming several times a day (vs. never or less than weekly) were less likely to report high life satisfaction, happiness, competence and relatedness. Boys who reported online gaming once per day (vs. never or less than weekly) were less likely to report high life satisfaction, happiness and relatedness, and boys who report online gaming weekly (vs. never or less than weekly) were less likely to report high life satisfaction and happiness (see Table 2).

Girls who reported online gaming constantly (vs. never or less than weekly) were less likely to report high happiness, autonomy, competence and relatedness. In addition, girls who reported online gaming several times a day or weekly (vs. never or less than weekly) were less likely to report high autonomy and competence (see Table 3).

### Screen time and positive mental health

Adjusted associations between specific ST behaviours (i.e. watching content and playing video games) and PMH outcomes are reported in Table 4 for boys and Table 5 for girls; unadjusted associations are reported in Table S3 for boys and Table S4 for girls in the Supplementary File. Adjusted associations between overall sedentary electronic device usage and PMH outcomes are reported in Table 6 for boys and Table 7 for girls; unadjusted associations are reported in Table S5 for boys and Table S6 for girls in the Supplementary File. As previously, we restrict our focus to associations that were statistically significant in the adjusted analyses.

**Table 4:** Adjusted associations between specific screen time behaviours and positive mental health among boys (*N* = 5,299)

	High self-rated mental health		High life satisfaction		High happiness		High autonomy		High competence		High relatedness	
	% (95% CI)	aOR (95% CI)	% (95% CI)	aOR (95% CI)	% (95% CI)	aOR (95% CI)	% (95% CI)	aOR (95% CI)	% (95% CI)	aOR (95% CI)	% (95% CI)	aOR (95% CI)
**Hours watching content in past week**												
< 3	81.6 (78.0, 85.1)	Ref.	62.1 (57.6, 66.6)	Ref.	76.7 (72.8, 80.6)	Ref.	84.3 (81.1, 87.4)	Ref.	88.9 (86.1, 91.7)	Ref.	91.3 (88.8, 93.7)	Ref.
3 to < 7	75.8 (72.9, 78.6)	**0.72** **(0.54**,** 0.97)**	52.7 (49.3, 56.0)	**0.73** **(0.57**,** 0.94)**	68.9 (65.7, 72.2)	**0.70** **(0.53**,** 0.92)**	83.6 (81.3, 86.0)	0.97 (0.71, 1.33)	86.9 (84.6, 89.2)	0.88 (0.61, 1.27)	90.1 (88.1, 92.2)	0.90 (0.61, 1.34)
7 to < 14	72.5 (69.4, 75.6)	**0.64** **(0.47**,** 0.86)**	48.1 (44.5, 51.7)	**0.65** **(0.50**,** 0.83)**	70.1 (66.9, 73.4)	0.85 (0.64, 1.12)	81.6 (79.0, 84.2)	0.89 (0.65, 1.22)	86.9 (84.5, 89.2)	0.98 (0.68, 1.41)	90.4 (88.3, 92.4)	1.04 (0.69, 1.56)
14 to < 21	71.0 (67.1, 75.0)	**0.62** **(0.45**,** 0.86)**	39.5 (35.2, 43.7)	**0.49** **(0.37**,** 0.65)**	63.8 (59.8, 67.9)	**0.70** **(0.52**,** 0.94)**	78.1 (74.5, 81.8)	0.75 (0.54, 1.06)	81.2 (77.5, 85.0)	0.73 (0.49, 1.10)	85.4 (82.3, 88.5)	0.74 (0.49, 1.12)
21+	67.1 (62.7, 71.5)	**0.55** **(0.38**,** 0.78)**	39.4 (34.8, 44.1)	**0.51** **(0.37**,** 0.70)**	58.5 (53.9, 63.0)	**0.67** **(0.48**,** 0.93)**	78.1 (74.4, 81.8)	0.79 (0.56, 1.12)	79.6 (75.6, 83.6)	0.75 (0.49, 1.15)	86.8 (83.7, 90.0)	1.01 (0.64, 1.60)
**Hours playing video games in past week**												
< 3	74.6 (71.9, 77.3)	Ref.	55.7 (52.5, 58.9)	Ref.	74.6 (71.7, 77.4)	Ref.	85.1 (83.0, 87.3)	Ref.	89.3 (87.4, 91.2)	Ref.	91.8 (90.1, 93.4)	Ref.
3 to < 7	75.3 (72.3, 78.4)	1.04 (0.83, 1.31)	47.5 (43.9, 51.1)	**0.72** **(0.59**,** 0.87)**	71.5 (68.2, 74.9)	0.87 (0.69, 1.09)	81.8 (79.1, 84.6)	0.80 (0.62, 1.03)	86.4 (83.6, 89.1)	0.74 (0.55, 1.02)	89.6 (87.3, 91.9)	0.77 (0.55, 1.08)
7 to < 14	74.3 (71.1, 77.5)	1.03 (0.82, 1.31)	48.0 (44.3, 51.7)	**0.78** **(0.63**,** 0.97)**	67.2 (63.6, 70.9)	**0.71** **(0.57**,** 0.89)**	81.8 (78.8, 84.7)	0.81 (0.61, 1.06)	85.1 (82.2, 87.9)	**0.68** **(0.50**,** 0.94)**	90.3 (88.0, 92.6)	0.84 (0.58, 1.21)
14 to < 21	73.5 (69.2, 77.8)	1.04 (0.79, 1.38)	43.3 (38.1, 48.6)	**0.72** **(0.55**,** 0.94)**	60.6 (55.5, 65.8)	**0.55** **(0.42**,** 0.72)**	75.7 (71.4, 79.9)	**0.59** **(0.43**,** 0.80)**	80.3 (75.9, 84.8)	**0.52** **(0.36**,** 0.75)**	85.6 (82.0, 89.2)	**0.56** **(0.38**,** 0.84)**
21+	68.5 (63.8, 73.1)	0.89 (0.66, 1.19)	41.4 (36.0, 46.8)	**0.70** **(0.52**,** 0.93)**	52.9 (47.5, 58.2)	**0.42** **(0.31**,** 0.56)**	77.0 (72.8, 81.2)	**0.64** **(0.47**,** 0.88)**	77.5 (73.1, 81.8)	**0.47** **(0.33**,** 0.67)**	82.3 (78.2, 86.5)	**0.43** **(0.29**,** 0.66)**

**Source**: 2019 Canadian Health Survey on Children and Youth

**Abbreviations**: aOR, adjusted odds ratio; CI, confidence interval; Ref., reference group

**Notes**: Sample size is unweighted; all other values are weighted. Statistically significant results are bolded. The adjusted logistic regression analyses included age, household income quintile, place of residence, population group and the other screen time variable as covariates

**Table 5:** Adjusted associations between specific screen time behaviours and positive mental health among girls (*N* = 5,396)

	High self-rated mental health		High life satisfaction		High happiness		High autonomy		High competence		High relatedness	
	% (95% CI)	aOR (95% CI)	% (95% CI)	aOR (95% CI)	% (95% CI)	aOR (95% CI)	% (95% CI)	aOR (95% CI)	% (95% CI)	aOR (95% CI)	% (95% CI)	aOR (95% CI)
**Hours watching content in past week**												
< 3	70.4 (66.2, 74.5)	Ref.	54.1 (49.6, 58.5)	Ref.	68.9 (64.8, 73.0)	Ref.	86.7 (84.1, 89.4)	Ref.	91.4 (89.1, 93.6)	Ref.	94.7 (93.0, 96.4)	Ref.
3 to < 7	64.3 (61.3, 67.2)	**0.77** **(0.60**,** 0.99)**	46.0 (42.7, 49.3)	**0.74** **(0.58**,** 0.93)**	64.4 (61.4, 67.5)	0.83 (0.65, 1.06)	82.7 (80.3, 85.0)	**0.75** **(0.56**,** 0.99)**	86.2 (83.8, 88.6)	**0.59** **(0.42**,** 0.84)**	91.7 (89.9, 93.5)	**0.60** **(0.39**,** 0.92)**
7 to < 14	56.3 (52.8, 59.8)	**0.59** **(0.46**,** 0.77)**	39.4 (35.9, 42.9)	**0.62** **(0.48**,** 0.78)**	59.5 (55.8, 63.1)	**0.72** **(0.56**,** 0.94)**	79.7 (76.8, 82.5)	**0.64** **(0.48**,** 0.86)**	82.9 (80.1, 85.8)	**0.48** **(0.33**,** 0.70)**	89.8 (87.4, 92.2)	**0.49** **(0.31**,** 0.76)**
14 to < 21	48.8 (43.9, 53.6)	**0.47** **(0.35**,** 0.63)**	29.3 (24.8, 33.8)	**0.42** **(0.31**,** 0.57)**	53.7 (48.8, 58.6)	**0.61** **(0.46**,** 0.81)**	75.7 (71.7, 79.7)	**0.53** **(0.38**,** 0.73)**	76.5 (72.1, 80.8)	**0.34** **(0.23**,** 0.50)**	85.4 (82.2, 88.6)	**0.34** **(0.22**,** 0.53)**
21+	46.3 (40.5, 52.1)	**0.45** **(0.32**,** 0.62)**	28.4 (23.1, 33.6)	**0.43** **(0.31**,** 0.60)**	48.4 (42.6, 54.1)	**0.52** **(0.38**,** 0.72)**	70.1 (64.8, 75.5)	**0.42** **(0.29**,** 0.61)**	73.1 (67.9, 78.4)	**0.30** **(0.20**,** 0.46)**	81.3 (76.7, 85.8)	**0.27** **(0.16**,** 0.44)**
**Hours playing video games in past week**												
< 3	59.9 (57.8, 62.1)	Ref.	43.3 (41.2, 45.5)	Ref.	63.6 (61.6, 65.6)	Ref.	82.0 (80.4, 83.7)	Ref.	85.1 (83.5, 86.7)	Ref.	89.9 (88.6, 91.3)	Ref.
3 to < 7	60.1 (55.4, 64.8)	0.95 (0.74, 1.20)	37.9 (33.0, 42.9)	**0.73** **(0.57**,** 0.94)**	56.4 (51.5, 61.3)	**0.68** **(0.54**,** 0.86)**	79.4 (75.6, 83.2)	0.84 (0.65, 1.10)	83.0 (79.2, 86.9)	0.86 (0.63, 1.17)	89.7 (86.7, 92.7)	0.97 (0.68, 1.39)
7 to < 14	55.4 (48.6, 62.2)	0.84 (0.63, 1.14)	38.1 (31.0, 45.2)	0.79 (0.58, 1.08)	51.9 (45.0, 58.7)	**0.59** **(0.44**,** 0.79)**	72.8 (66.6, 79.0)	**0.63** **(0.44**,** 0.89)**	74.1 (67.7, 80.5)	**0.55** **(0.38**,** 0.79)**	89.7 (86.1, 93.4)	1.07 (0.69, 1.64)
14 to < 21	52.7 (42.7, 62.7)	0.74 (0.47, 1.18)	28.7^C^ (20.0, 37.4)	**0.51** **(0.32**,** 0.80)**	42.1 (32.4, 51.8)	**0.39** **(0.25**,** 0.61)**	69.8 (60.9, 78.7)	**0.58** **(0.36**,** 0.95)**	75.6 (67.1, 84.0)	0.62 (0.36, 1.05)	85.2 (79.4, 91.0)	0.74 (0.42, 1.29)
21+	44.2 (33.9, 54.5)	0.65 (0.42, 1.01)	27.8^C^ (18.8, 36.7)	**0.60** **(0.37**,** 0.97)**	50.0 (39.5, 60.5)	0.66 (0.42, 1.03)	68.9 (59.4, 78.4)	0.64 (0.39, 1.06)	73.2 (64.3, 82.1)	0.65 (0.39, 1.10)	86.1 (79.7, 92.5)	1.00 (0.52, 1.91)

**Source**: 2019 Canadian Health Survey on Children and Youth

**Abbreviations**: aOR, adjusted odds ratio; CI, confidence interval; Ref., reference group

**Notes**: Sample size is unweighted; all other values are weighted. Statistically significant results are bolded. The adjusted logistic regression analyses included age, household income quintile, place of residence, population group and the other screen time variable as covariates

^C^ Estimate should be interpreted with caution due to high sampling variability

**Table 6:** Adjusted associations between overall sedentary electronic device usage and positive mental health among boys (*N* = 5,299)

	High self-rated mental health		High life satisfaction		High happiness		High autonomy		High competence		High relatedness	
	% (95% CI)	aOR (95% CI)	% (95% CI)	aOR (95% CI)	% (95% CI)	aOR (95% CI)	% (95% CI)	aOR (95% CI)	% (95% CI)	aOR (95% CI)	% (95% CI)	aOR (95% CI)
**Hours of electronic device use in past week**												
< 3	77.2 (72.4, 82.1)	Ref.	62.4 (56.9, 68.0)	Ref.	80.4 (75.9, 84.9)	Ref.	85.9 (82.2, 89.5)	Ref.	92.0 (89.3, 94.7)	Ref.	93.7 (91.3, 96.2)	Ref.
3 to < 7	78.3 (75.0, 81.6)	1.09 (0.77, 1.56)	56.2 (52.3, 60.1)	0.81 (0.60, 1.08)	71.7 (68.0, 75.4)	**0.63** **(0.45**,** 0.90)**	83.9 (81.0, 86.7)	0.84 (0.58, 1.22)	87.7 (85.1, 90.4)	**0.62** **(0.40**,** 0.98)**	90.0 (87.6, 92.4)	0.61 (0.36, 1.02)
7 to < 14	75.6 (72.5, 78.7)	0.96 (0.70, 1.33)	52.6 (48.8, 56.4)	**0.71** **(0.53**,** 0.94)**	74.7 (71.6, 77.8)	0.75 (0.54, 1.04)	81.6 (78.7, 84.6)	0.71 (0.50, 1.02)	87.8 (85.1, 90.5)	0.64 (0.41, 1.02)	91.0 (88.9, 93.1)	0.69 (0.41, 1.16)
14 to < 21	73.1 (69.6, 76.6)	0.86 (0.62, 1.20)	43.5 (39.5, 47.5)	**0.50** **(0.38**,** 0.67)**	64.8 (60.9, 68.6)	**0.47** **(0.34**,** 0.66)**	81.3 (78.4, 84.3)	0.70 (0.48, 1.01)	85.2 (82.2, 88.1)	**0.51** **(0.32**,** 0.80)**	90.9 (88.7, 93.2)	0.70 (0.42, 1.17)
21+	68.1 (64.9, 71.3)	**0.69** **(0.50**,** 0.96)**	39.4 (35.8, 43.1)	**0.44** **(0.33**,** 0.58)**	57.8 (54.3, 61.3)	**0.36** **(0.26**,** 0.49)**	78.2 (75.5, 81.0)	**0.57** **(0.41**,** 0.80)**	78.6 (75.5, 81.7)	**0.33** **(0.22**,** 0.51)**	83.4 (80.7, 86.1)	**0.35** **(0.22**,** 0.58)**

**Source**: 2019 Canadian Health Survey on Children and Youth

**Abbreviations**: aOR, adjusted odds ratio; CI, confidence interval; Ref., reference group

**Notes**: Sample size is unweighted; all other values are weighted. Statistically significant results are bolded. The adjusted logistic regression analyses included age, household income quintile, place of residence and population group as covariates

**Table 7:** Adjusted associations between overall sedentary electronic device usage and positive mental health among girls (*N* = 5,396)

	High self-rated mental health		High life satisfaction		High happiness		High autonomy		High competence		High relatedness	
	% (95% CI)	aOR (95% CI)	% (95% CI)	aOR (95% CI)	% (95% CI)	aOR (95% CI)	% (95% CI)	aOR (95% CI)	% (95% CI)	aOR (95% CI)	% (95% CI)	aOR (95% CI)
**Hours of electronic device use in past week**												
< 3	70.0 (65.1, 75.0)	Ref.	58.2 (52.8, 63.5)	Ref.	75.8 (71.1, 80.4)	Ref.	88.2 (84.8, 91.5)	Ref.	92.8 (90.0, 95.5)	Ref.	95.6 (93.4, 97.8)	Ref.
3 to < 7	66.8 (63.3, 70.3)	0.93 (0.69, 1.24)	49.4 (45.6, 53.3)	**0.74** **(0.56**,** 0.98)**	68.6 (65.2, 72.0)	**0.70** **(0.52**,** 0.95)**	84.6 (81.9, 87.2)	0.76 (0.52, 1.13)	87.8 (85.3, 90.3)	**0.57** **(0.36**,** 0.93)**	91.7 (89.4, 93.9)	**0.53** **(0.29**,** 0.98)**
7 to < 14	60.4 (57.0, 63.8)	0.75 (0.56, 1.00002)	41.3 (37.6, 44.9)	**0.56** **(0.43**,** 0.74)**	62.1 (58.6, 65.6)	**0.53** **(0.39**,** 0.72)**	80.3 (77.4, 83.2)	**0.56** **(0.38**,** 0.82)**	84.2 (81.3, 87.1)	**0.42** **(0.26**,** 0.68)**	90.8 (88.7, 92.8)	**0.48** **(0.26**,** 0.87)**
14 to < 21	54.0 (50.0, 58.0)	**0.59** **(0.44**,** 0.80)**	37.2 (33.2, 41.3)	**0.49** **(0.37**,** 0.66)**	56.2 (52.0, 60.3)	**0.43** **(0.31**,** 0.58)**	78.0 (74.6, 81.3)	**0.50** **(0.34**,** 0.74)**	81.2 (78.0, 84.4)	**0.35** **(0.22**,** 0.56)**	88.3 (85.8, 90.7)	**0.38** **(0.21**,** 0.68)**
21+	46.3 (41.9, 50.7)	**0.45** **(0.33**,** 0.61)**	25.8 (22.0, 29.6)	**0.30** **(0.22**,** 0.40)**	44.7 (40.4, 49.1)	**0.28** **(0.20**,** 0.38)**	72.3 (68.4, 76.2)	**0.38** **(0.25**,** 0.56)**	73.6 (69.6, 77.7)	**0.24** **(0.15**,** 0.38)**	83.7 (80.4, 87.0)	**0.27** **(0.15**,** 0.50)**

**Source**: 2019 Canadian Health Survey on Children and Youth

**Abbreviations**: aOR, adjusted odds ratio; CI, confidence interval; Ref., reference group

**Notes**: Sample size is unweighted; all other values are weighted. Statistically significant results are bolded. The adjusted logistic regression analyses included age, household income quintile, place of residence and population group as covariates

#### Hours spent watching content

Boys who reported watching content for 14 + hours in the past week (vs. < 3 h) were less likely to report high SRMH, life satisfaction and happiness. Moreover, boys who reported watching content between 7 and 14 h (vs. < 3) were less likely to report high SRMH and life satisfaction, while boys who reported watching content between 3 and 7 h (vs. < 3) were less likely to report high SRMH, life satisfaction and happiness (see Table 4).

Girls who reported watching content for 7 + hours in the past week (vs. < 3 h) were less likely to report high PMH across all six outcomes. In addition, girls who reported watching content between 3 and 7 h (vs. < 3) were less likely to report high SRMH, life satisfaction, autonomy, competence and relatedness (see Table 5).

#### Hours spent playing video games

Boys who reported playing video games 14 + hours in the past week (vs. < 3 h) were less likely to report high life satisfaction, happiness, autonomy, competence and relatedness. In addition, boys who reported playing video games between 7 and 14 h (vs. < 3) were less likely to report high life satisfaction, happiness and competence, while boys who reported playing video games 3–7 h (vs. < 3) were less likely to report high life satisfaction (see Table 4).

Girls who reported playing video games for 21 + hours in the past week (vs. < 3 h) were less likely to report high life satisfaction. Girls who reported playing video games between 14 and 21 h (vs. < 3) were less likely to report high life satisfaction, happiness and autonomy. Girls who reported playing video games between 7 and 14 h (vs. < 3) were less likely to report high happiness, autonomy and competence. Lastly, girls who reported playing video games between 3 and 7 h (vs. < 3) were less likely to report high life satisfaction and happiness (see Table 5).

#### Hours spent sedentary on electronic devices

Boys who reported 21 + hours of sedentary electronic device usage in the past week (vs. < 3 h) were less likely to report high PMH across all six outcomes. Boys who reported between 14 and 21 h of sedentary electronic device usage (vs. < 3) were also less likely to report high life satisfaction, happiness and competence. Boys who reported between 7 and 14 h of sedentary electronic device usage (vs. < 3) were less likely to report high life satisfaction, while those who reported between 3 and 7 h (vs. < 3) were less likely to report high happiness and competence (see Table 6).

Girls who reported 14–21 h of sedentary electronic device usage in the past week (vs. < 3 h) were less likely to report high PMH across all six outcomes. Furthermore, girls who reported 7–14 h of sedentary electronic device usage (vs. < 3) were less likely to report high life satisfaction, happiness, autonomy, competence and relatedness. Lastly, girls who reported between 3 and 7 h of sedentary electronic device usage (vs. < 3) were also less likely to report high life satisfaction, happiness, competence and relatedness (see Table 7).

## Discussion

This study examined associations between DMU, ST and PMH indicators using 2019 data from over 10,000 youth in Canada. DMU and ST were a regular part of youth’s daily lives, and we found some evidence for their link with PMH. Nevertheless, reinforcing the nuances and inconsistencies in the broader literature, [3, 15, 16, 17, 18, 19, 20, 21, 22, 23, 24] associations with PMH were not ubiquitous and appeared to depend on several factors.

We found that SMU was more consistently associated with lower odds of PMH among girls than boys. This aligns with some research showing stronger associations between SMU and internalizing symptoms for girls than boys [32]. Moreover, SMU was found to be more robustly related to lower satisfaction with different life domains among UK girls than boys, [18] and high (vs. light) SMU was more strongly linked to unhappiness among US girls than boys [33]. The latter study also found that light (vs. no) SMU was associated with higher happiness for boys but not girls [33]. Our analyses also revealed higher levels of happiness and/or autonomy among boys who engaged in some SMU (i.e., once or several times a day) compared to boys who did not or did so rarely (i.e., never or less than weekly). Various potential explanations have been provided for the stronger relationship between SMU and negative psychological outcomes observed among girls, such as greater susceptibility to upward social comparison, online harassment, and body dissatisfaction [32]. Future research could explore these as possible mechanisms for SMU links with PMH among Canadian youth.

In contrast, video/instant messaging was not associated with lower odds of PMH for girls or boys in any of our adjusted analyses and was even positively associated with at least one PMH outcome for boys who reported video/instant messaging weekly, daily or several times a day (vs. never or less than weekly). Other research using 2019 CHSCY data reported that constant video/instant messaging was associated with some poorer mental health outcomes, even after adjusting for sociodemographic factors [2]. However, that study examined each DMU behaviour separately, while we controlled for other DMU behaviours in adjusted analyses. Negative associations involving video/instant messaging and PMH in some of our unadjusted analyses may have been partially due to engagement in other correlated DMU behaviours like SMU [34]. Video/instant messaging may be less detrimental (and even beneficial in certain instances) for PMH as it can provide opportunities to develop social skills, express emotions, self-disclose and strengthen/maintain interpersonal relationships [35, 36, 37, 38]. Video/instant messaging shares more features of ‘real world’ social interactions than SMU as it can be synchronous and involve one-to-one or one-to-several communication (vs. one-to-many communication) [39]. Like our study, some research suggests that this form of online communication may be particularly beneficial for boys [35, 38]. The role of video/instant messaging in the PMH of Canadian youth during the COVID-19 pandemic when in-person communication was limited could be explored in future research.

For both boys and girls, reports of constant online gaming (vs. never or less than weekly) were associated with lower odds of most PMH outcomes. This may reflect more addiction-like tendencies towards gaming. Meta-analytic research finds that internet gaming disorder symptoms are positively correlated with psychological and interpersonal problems, and negatively correlated with individual well-being, although the magnitude/presence of these associations appear to vary cross-culturally [40]. Online gaming could also displace other activities that are beneficial for PMH, including health behaviours such as physical activity [26, 41, 42].

In terms of ST, we tended to observe more consistent associations across PMH outcomes at greater levels of ST exposure. Time spent playing video games among girls was an exception: those with the greatest level of exposure were only significantly lower on one PMH outcome in the adjusted analyses (albeit they were lower on almost all outcomes in the unadjusted analysis). Nevertheless, the threshold for ST being linked to lower PMH could be quite minimal. For instance, boys and girls who reported as little as 3–7 h per week of overall sedentary electronic device usage, which could be considered meeting ST recommendations, [4] had lower odds of at least two PMH outcomes compared to those who reported under 3 h of usage per week. Relatedly, we did not find evidence for the goldilocks hypothesis for ST as moderate amounts were not linked to higher PMH, [21, 22] although our combination of “No time” and “Less than 3 hours” for the reference group limits more definitive conclusions. Negative associations with PMH also tended to be more consistent for girls than boys for time spent watching content and overall sedentary electronic device usage. Future studies could explore how gender differences in the type of content watched and preferred activities on electronic devices might play a role in the differing patterns of results.

### Strengths and limitations

The 2019 CHSCY is a large population-based survey, which afforded sufficient statistical power to investigate PMH by detailed levels of DMU and ST, adjust for a range of potentially confounding variables and stratify results by gender. In addition, we were able to examine associations between a breadth of DMU, ST and PMH indicators, allowing us to address important evidence gaps.

Regarding limitations, the use of cross-sectional data means we cannot ascertain causality. DMU and ST were self-reported, which can be inaccurate [43, 44]. Response options for DMU and ST differed, making it difficult to compare their associations. There is also overlap in some of the activities we examined. For instance, time spent playing video games could include online gaming, but also other formats like local multiplayer that involve in-person social interaction. How different formats or genres of video games, and motivations for playing video games are associated with PMH among Canadian youth could be explored in future studies [45]. Greater specificity for the other DMU and ST activities could also be informative (e.g., active or passive SMU; the type of content being watched) [46]. Different reference groups could have been chosen for the DMU and ST variables, and there is the potential for residual confounding. Finally, results may not generalize to groups who were excluded from CHSCY or from analyses. For instance, non-binary youth were not examined in the current study; research from Europe has found that these youth report spending more time online, engage in DMU differently and have lower levels of PMH compared to boys and girls [47].

## Conclusion

DMU behaviours and ST are prevalent and can be associated with lower odds of PMH outcomes among boys and girls in Canada. Longitudinal study designs are needed to better ascertain the causal direction of these associations. In addition, observed associations may have changed over time; there are indications of some PMH outcomes decreasing at the population level, as well as fewer youth meeting ST recommendations, during the COVID-19 pandemic [7, 26]. Fortunately, a longitudinal follow-up involving 2019 CHSCY respondents was conducted in 2023 that could be used to fill these evidence gaps [48]. Results can assist in the promotion of public health strategies targeted towards promoting reduced and/or non-problematic DMU and ST, and improved well-being, as well as the continued surveillance of DMU, ST and PMH outcomes among Canadian youth.

## Electronic supplementary material

Below is the link to the electronic supplementary material.

Supplementary Material 1

## Data Availability

The questionnaire is available on this Statistics Canada webpage: https://www23.statcan.gc.ca/imdb/p3Instr.pl?Function=getInstrumentList%26Item_Id=1209093%26UL=1V. The data are available at Research Data Centres: https://www.statcan.gc.ca/en/microdata/data-centres.

## Abbreviations

CHSCY: Canadian Health Survey on Children and Youth
CI: Confidence interval
DMU: Digital media use
PMH: Positive mental health
SMU: Social media use
SRMH: Self-rated mental health
ST: Screen time
UK: United Kingdom
US: United States
