# Supplementary material for: Associations between digital media use behaviours, screen time and positive mental health in youth: results from the 2019 Canadian Health Survey on Children and Youth
Source: BMC Public Health. 2025 Jul 3;25:2303. doi: 10.1186/s12889-025-22874-2 (PMC12225355; doi:10.1186/s12889-025-22874-2)
Supplement: Supplementary file 1 — Supplementary Material 1 [file 12889_2025_22874_MOESM1_ESM.docx]

Supplementary File

**Associations between digital media use behaviours, screen time and positive mental health in youth: results from the 2019 Canadian Health Survey on Children and Youth**

**Table S1. Unadjusted associations between digital media use behaviours and positive mental health among boys (*N* = 5,299)**

|  | **High self-rated  mental health** | **High life  satisfaction** | **High  happiness** | **High  autonomy** | **High  competence** | **High  relatedness** |
| --- | --- | --- | --- | --- | --- | --- |
|  | OR (95% CI) | OR (95% CI) | OR (95% CI) | OR (95% CI) | OR (95% CI) | OR (95% CI) |
| **Social media** |  |  |  |  |  |  |
| Never or less than weekly | Ref. | Ref. | Ref. | Ref. | Ref. | Ref. |
| Weekly | 0.99  (0.68, 1.44) | 1.02  (0.73, 1.43) | 0.88  (0.61, 1.26) | 0.89  (0.58, 1.37) | 0.72  (0.45, 1.18) | 0.81  (0.45, 1.46) |
| Once a day | 1.11  (0.85, 1.44) | 0.91  (0.72, 1.14) | 1.09  (0.85, 1.39) | **1.55**  **(1.13, 2.13)** | 1.05  (0.73, 1.53) | 0.85  (0.58, 1.25) |
| Several times a day | 0.84  (0.69, 1.03) | 0.87  (0.73, 1.04) | 1.14  (0.94, 1.38) | **1.39**  **(1.12, 1.72)** | 0.98  (0.76, 1.26) | 0.98  (0.74, 1.29) |
| Constantly | **0.76**  **(0.59, 0.99)** | **0.77**  **(0.59, 0.996)** | 0.91  (0.70, 1.18) | 1.13  (0.84, 1.52) | **0.68**  **(0.48, 0.96)** | **0.66**  **(0.46, 0.96)** |
| **Video / instant messaging** |  |  |  |  |  |  |
| Never or less than weekly | Ref. | Ref. | Ref. | Ref. | Ref. | Ref. |
| Weekly | **1.59**  **(1.11, 2.26)** | 1.21  (0.89, 1.65) | 1.13  (0.79, 1.61) | **1.68**  **(1.13, 2.51)** | 1.20  (0.72, 2.02) | 1.12  (0.66, 1.93) |
| Once a day | 1.20  (0.93, 1.54) | 1.09  (0.87, 1.37) | 1.23  (0.96, 1.57) | **1.43**  **(1.08, 1.91)** | 1.05  (0.74, 1.48) | 1.46  (0.998, 2.13) |
| Several times a day | 0.97  (0.80, 1.19) | 0.92  (0.77, 1.11) | 1.16  (0.95, 1.41) | **1.37**  **(1.10, 1.70)** | 1.05  (0.81, 1.37) | 1.07  (0.81, 1.42) |
| Constantly | **0.69**  **(0.54, 0.88)** | **0.71**  **(0.56, 0.91)** | 0.91  (0.71, 1.15) | 1.08  (0.82, 1.42) | 0.77  (0.56, 1.05) | 0.82  (0.58, 1.15) |
| **Online gaming** |  |  |  |  |  |  |
| Never or less than weekly | Ref. | Ref. | Ref. | Ref. | Ref. | Ref. |
| Weekly | 1.09  (0.84, 1.41) | 0.80  (0.64, 1.003) | **0.74**  **(0.57, 0.95)** | 1.03  (0.77, 1.38) | 0.80  (0.57, 1.11) | 0.71  (0.48, 1.05) |
| Once a day | 1.18  (0.94, 1.49) | 0.82  (0.67, 1.01) | **0.80**  **(0.63, 0.999)** | 1.12  (0.86, 1.47) | 0.82  (0.60, 1.12) | 0.75  (0.55, 1.04) |
| Several times a day | 1.00  (0.80, 1.24) | **0.79**  **(0.64, 0.97)** | **0.55**  **(0.45, 0.69)** | 0.87  (0.68, 1.11) | **0.61**  **(0.46, 0.82)** | **0.67**  **(0.49, 0.92)** |
| Constantly | 0.79  (0.60, 1.04) | **0.67**  **(0.51, 0.88)** | **0.42**  **(0.32, 0.55)** | **0.53**  **(0.40, 0.70)** | **0.47**  **(0.33, 0.66)** | **0.38**  **(0.27, 0.56)** |

**Source:** 2019 Canadian Health Survey on Children and Youth. **Abbreviations:** CI, confidence interval; OR, odds ratio; Ref., reference group.

**Notes:** Sample size is unweighted; all other values are weighted. Statistically significant results are bolded.

**Table S2. Unadjusted associations between digital media use behaviours and positive mental health among girls (*N* = 5,396)**

|  | **High self-rated  mental health** | **High life  satisfaction** | **High  happiness** | **High  autonomy** | **High  competence** | **High  relatedness** |
| --- | --- | --- | --- | --- | --- | --- |
|  | OR (95% CI) | OR (95% CI) | OR (95% CI) | OR (95% CI) | OR (95% CI) | OR (95% CI) |
| **Social media** |  |  |  |  |  |  |
| Never or less than weekly | Ref. | Ref. | Ref. | Ref. | Ref. | Ref. |
| Weekly | 0.74  (0.46, 1.17) | **0.50**  **(0.32, 0.77)** | **0.47**  **(0.30, 0.76)** | 0.83  (0.49, 1.41) | 0.69  (0.37, 1.29) | 0.71  (0.33, 1.52) |
| Once a day | **0.62**  **(0.46, 0.84)** | **0.61**  **(0.45, 0.81)** | **0.66**  **(0.48, 0.91)** | 0.77  (0.53, 1.12) | 0.73  (0.47, 1.15) | 0.64  (0.37, 1.11) |
| Several times a day | **0.50**  **(0.40, 0.62)** | **0.37**  **(0.30, 0.46)** | **0.48**  **(0.38, 0.61)** | 0.81  (0.61, 1.07) | **0.62**  **(0.43, 0.89)** | **0.57**  **(0.37, 0.87)** |
| Constantly | **0.35**  **(0.27, 0.44)** | **0.29**  **(0.22, 0.37)** | **0.39**  **(0.30, 0.51)** | **0.59**  **(0.43, 0.80)** | **0.47**  **(0.32, 0.68)** | **0.43**  **(0.27, 0.68)** |
| **Video / instant messaging** |  |  |  |  |  |  |
| Never or less than weekly | Ref. | Ref. | Ref. | Ref. | Ref. | Ref. |
| Weekly | 0.71  (0.51, 1.003) | 0.75  (0.53, 1.06) | 0.94  (0.65, 1.34) | 1.02  (0.64, 1.62) | 1.04  (0.59, 1.81) | 0.85  (0.46, 1.55) |
| Once a day | 0.78  (0.59, 1.02) | **0.67**  **(0.52, 0.87)** | 0.98  (0.75, 1.29) | 1.06  (0.77, 1.46) | 1.08  (0.74, 1.58) | 0.81  (0.51, 1.27) |
| Several times a day | **0.64**  **(0.52, 0.79)** | **0.58**  **(0.47, 0.71)** | **0.76**  **(0.61, 0.94)** | 0.94  (0.74, 1.20) | 0.91  (0.69, 1.21) | 0.78  (0.55, 1.10) |
| Constantly | **0.52**  **(0.41, 0.67)** | **0.49**  **(0.38, 0.62)** | **0.62**  **(0.49, 0.79)** | 0.87  (0.66, 1.14) | **0.70**  **(0.51, 0.96)** | **0.68**  **(0.46, 0.99)** |
| **Online gaming** |  |  |  |  |  |  |
| Never or less than weekly | Ref. | Ref. | Ref. | Ref. | Ref. | Ref. |
| Weekly | 0.84  (0.62, 1.14) | 0.80  (0.59, 1.09) | 0.77  (0.57, 1.04) | **0.63**  **(0.43, 0.91)** | **0.60**  **(0.40, 0.92)** | 0.63  (0.38, 1.03) |
| Once a day | **1.42**  **(1.02, 1.97)** | 1.20  (0.87, 1.65) | 0.91  (0.66, 1.27) | 1.07  (0.71, 1.63) | 0.76  (0.48, 1.19) | 0.72  (0.40, 1.30) |
| Several times a day | 1.00  (0.69, 1.47) | 0.80  (0.55, 1.17) | 0.73  (0.50, 1.08) | **0.60**  **(0.39, 0.92)** | **0.49**  **(0.32, 0.75)** | 0.72  (0.40, 1.30) |
| Constantly | 0.86  (0.53, 1.38) | **0.58**  **(0.34, 0.97)** | **0.46**  **(0.28, 0.75)** | **0.44**  **(0.27, 0.72)** | **0.36**  **(0.21, 0.60)** | **0.33**  **(0.19, 0.59)** |

**Source:** 2019 Canadian Health Survey on Children and Youth. **Abbreviations:** CI, confidence interval; OR, odds ratio; Ref., reference group.

**Notes:** Sample size is unweighted; all other values are weighted. Statistically significant results are bolded.

**Table S3. Unadjusted associations between specific screen time behaviours and positive mental health among boys (*N* = 5,299)**

|  | **High self-rated mental health** | **High life  satisfaction** | **High happiness** | **High  autonomy** | **High  competence** | **High  relatedness** |
| --- | --- | --- | --- | --- | --- | --- |
|  | OR (95% CI) | OR (95% CI) | OR (95% CI) | OR (95% CI) | OR (95% CI) | OR (95% CI) |
| **Hours watching content in past week** |  |  |  |  |  |  |
| <3 | Ref. | Ref. | Ref. | Ref. | Ref. | Ref. |
| 3 to <7 | **0.71**  **(0.53, 0.94)** | **0.68**  **(0.54, 0.86)** | **0.67**  **(0.52, 0.88)** | 0.96  (0.70, 1.30) | 0.83  (0.58, 1.18) | 0.87  (0.59, 1.29) |
| 7 to <14 | **0.60**  **(0.45, 0.79)** | **0.57**  **(0.45, 0.72)** | **0.71**  **(0.55, 0.93)** | 0.83  (0.62, 1.12) | 0.82  (0.58, 1.16) | 0.90  (0.61, 1.32) |
| 14 to <21 | **0.55**  **(0.41, 0.75)** | **0.40**  **(0.31, 0.52)** | **0.54**  **(0.41, 0.71)** | **0.67**  **(0.49, 0.92)** | **0.54**  **(0.37, 0.79)** | **0.56**  **(0.38, 0.83)** |
| 21+ | **0.46**  **(0.34, 0.63)** | **0.40**  **(0.30, 0.53)** | **0.43**  **(0.32, 0.57)** | **0.67**  **(0.49, 0.92)** | **0.49**  **(0.33, 0.71)** | **0.63**  **(0.41, 0.95)** |
| **Hours playing video games in past week** |  |  |  |  |  |  |
| <3 | Ref. | Ref. | Ref. | Ref. | Ref. | Ref. |
| 3 to <7 | 1.04  (0.83, 1.30) | 0.72  (0.59, 0.87) | 0.86  (0.68, 1.07) | 0.79  (0.61, 1.01) | 0.76  (0.56, 1.03) | 0.77  (0.55, 1.07) |
| 7 to <14 | 0.98  (0.79, 1.23) | **0.73**  **(0.60, 0.89)** | **0.70**  **(0.56, 0.87)** | 0.78  (0.60, 1.02) | **0.68**  **(0.51, 0.92)** | 0.83  (0.59, 1.18) |
| 14 to <21 | 0.94  (0.72, 1.23) | **0.61**  **(0.47, 0.78)** | **0.53**  **(0.40, 0.68)** | **0.54**  **(0.41, 0.72)** | **0.49**  **(0.35, 0.69)** | **0.53**  **(0.37, 0.78)** |
| 21+ | **0.74**  **(0.57, 0.96)** | **0.56**  **(0.43, 0.73)** | **0.38**  **(0.30, 0.50)** | **0.59**  **(0.44, 0.77)** | **0.41**  **(0.30, 0.57)** | **0.42**  **(0.29, 0.60)** |

**Source:** 2019 Canadian Health Survey on Children and Youth.

**Abbreviations:** CI, confidence interval; OR, odds ratio; Ref., reference group.

**Notes:** Sample size is unweighted; all other values are weighted. Statistically significant results are bolded.

**Table S4. Unadjusted associations between specific screen time behaviours and positive mental health among girls (*N* = 5,396)**

|  | **High self-rated mental health** | **High life  satisfaction** | **High happiness** | **High  autonomy** | **High  competence** | **High  relatedness** |
| --- | --- | --- | --- | --- | --- | --- |
|  | OR (95% CI) | OR (95% CI) | OR (95% CI) | OR (95% CI) | OR (95% CI) | OR (95% CI) |
| **Hours watching content in past week** |  |  |  |  |  |  |
| <3 | Ref. | Ref. | Ref. | Ref. | Ref. | Ref. |
| 3 to <7 | **0.76**  **(0.60, 0.96)** | **0.72**  **(0.58, 0.91)** | 0.82  (0.65, 1.04) | **0.73**  **(0.55, 0.97)** | **0.59**  **(0.42, 0.83)** | **0.62**  **(0.41, 0.94)** |
| 7 to <14 | **0.54**  **(0.42, 0.69)** | **0.55**  **(0.44, 0.70)** | **0.66**  **(0.52, 0.85)** | **0.60**  **(0.45, 0.81)** | **0.46**  **(0.32, 0.66)** | **0.49**  **(0.32, 0.76)** |
| 14 to <21 | **0.40**  **(0.30, 0.53)** | **0.35**  **(0.27, 0.47)** | **0.52**  **(0.40, 0.69)** | **0.48**  **(0.35, 0.65)** | **0.31**  **(0.21, 0.44)** | **0.33**  **(0.22, 0.50)** |
| 21+ | **0.36**  **(0.27, 0.50)** | **0.34**  **(0.25, 0.46)** | **0.42**  **(0.31, 0.57)** | **0.36**  **(0.26, 0.51)** | **0.26**  **(0.18, 0.38)** | **0.24**  **(0.15, 0.38)** |
| **Hours playing video games in past week** |  |  |  |  |  |  |
| <3 | Ref. | Ref. | Ref. | Ref. | Ref. | Ref. |
| 3 to <7 | 1.01  (0.81, 1.25) | 0.80  (0.64, 1.002) | **0.74**  **(0.59, 0.92)** | 0.84  (0.65, 1.09) | 0.86  (0.63, 1.16) | 0.98  (0.69, 1.39) |
| 7 to <14 | 0.83  (0.62, 1.11) | 0.81  (0.59, 1.10) | **0.62**  **(0.46, 0.82)** | **0.59**  **(0.42, 0.82)** | **0.50**  **(0.35, 0.72)** | 0.98  (0.65, 1.49) |
| 14 to <21 | 0.74  (0.49, 1.13) | **0.53**  **(0.34, 0.82)** | **0.42**  **(0.28, 0.63)** | **0.51**  **(0.33, 0.79)** | **0.54**  **(0.33, 0.88)** | 0.64  (0.39, 1.06) |
| 21+ | **0.53**  **(0.34, 0.82)** | **0.50**  **(0.32, 0.80)** | **0.57**  **(0.37, 0.88)** | **0.49**  **(0.31, 0.77)** | **0.48**  **(0.30, 0.77)** | 0.70  (0.39, 1.23) |

**Source:** 2019 Canadian Health Survey on Children and Youth.

**Abbreviations:** CI, confidence interval; OR, odds ratio; Ref., reference group.

**Notes:** Sample size is unweighted; all other values are weighted. Statistically significant results are bolded.

**Table S5. Unadjusted associations between overall sedentary electronic device usage and positive mental health among boys (*N* = 5,299)**

|  | **High self-rated  mental health** | **High life  satisfaction** | **High  happiness** | **High  autonomy** | **High  competence** | **High  relatedness** |
| --- | --- | --- | --- | --- | --- | --- |
|  | OR (95% CI) | OR (95% CI) | OR (95% CI) | OR (95% CI) | OR (95% CI) | OR (95% CI) |
| **Hours of electronic device use in past week** |  |  |  |  |  |  |
| <3 | Ref. | Ref. | Ref. | Ref. | Ref. | Ref. |
| 3 to <7 | 1.06 (0.75, 1.50) | 0.77 (0.58, 1.03) | **0.62 (0.44, 0.87)** | 0.86 (0.59, 1.24) | **0.62 (0.40, 0.97)** | 0.60 (0.36, 1.002) |
| 7 to <14 | 0.91 (0.67, 1.25) | **0.67 (0.50, 0.89)** | **0.72 (0.52, 0.996)** | 0.73 (0.51, 1.04) | **0.63 (0.40, 0.99)** | 0.68 (0.41, 1.13) |
| 14 to <21 | 0.80 (0.58, 1.11) | **0.46 (0.35, 0.62)** | **0.45 (0.32, 0.63)** | 0.72 (0.50, 1.04) | **0.50 (0.32, 0.78)** | 0.67 (0.40, 1.12) |
| 21+ | **0.63 (0.46, 0.86)** | **0.39 (0.30, 0.52)** | **0.33 (0.24, 0.46)** | **0.59 (0.42, 0.83)** | **0.32 (0.21, 0.48)** | **0.34 (0.21, 0.54)** |

**Source:** 2019 Canadian Health Survey on Children and Youth.

**Abbreviations:** CI, confidence interval; OR, odds ratio; Ref., reference group.

**Notes:** Sample size is unweighted; all other values are weighted. Statistically significant results are bolded.

**Table S6. Unadjusted associations between overall sedentary electronic device usage and positive mental health among girls (*N* = 5,396)**

|  | **High self-rated  mental health** | **High life  satisfaction** | **High  happiness** | **High  autonomy** | **High  competence** | **High  relatedness** |
| --- | --- | --- | --- | --- | --- | --- |
|  | OR (95% CI) | OR (95% CI) | OR (95% CI) | OR (95% CI) | OR (95% CI) | OR (95% CI) |
| **Hours of electronic device use in past week** |  |  |  |  |  |  |
| <3 | Ref. | Ref. | Ref. | Ref. | Ref. | Ref. |
| 3 to <7 | 0.86 (0.65, 1.14) | **0.70 (0.54, 0.92)** | **0.70 (0.52, 0.94)** | 0.73 (0.50, 1.08) | **0.56 (0.34, 0.90)** | **0.51 (0.28, 0.92)** |
| 7 to <14 | **0.65 (0.49, 0.86)** | **0.51 (0.39, 0.66)** | **0.52 (0.39, 0.71)** | **0.55 (0.37, 0.80)** | **0.42 (0.26, 0.67)** | **0.45 (0.25, 0.81)** |
| 14 to <21 | **0.50 (0.38, 0.67)** | **0.43 (0.32, 0.57)** | **0.41 (0.30, 0.56)** | **0.47 (0.32, 0.70)** | **0.34 (0.21, 0.54)** | **0.35 (0.19, 0.62)** |
| 21+ | **0.37 (0.28, 0.49)** | **0.25 (0.19, 0.34)** | **0.26 (0.19, 0.35)** | **0.35 (0.24, 0.52)** | **0.22 (0.14, 0.35)** | **0.24 (0.13, 0.43)** |

**Source:** 2019 Canadian Health Survey on Children and Youth.

**Abbreviations:** CI, confidence interval; OR, odds ratio; Ref., reference group.

**Notes:** Sample size is unweighted; all other values are weighted. Statistically significant results are bolded.
